# Prognostic significance of body temperature in the emergency department vs the ICU in Patients with severe sepsis or septic shock: A nationwide cohort study

**DOI:** 10.1371/journal.pone.0243990

**Published:** 2020-12-29

**Authors:** Malin Inghammar, Jonas Sunden-Cullberg

**Affiliations:** 1 Department of Clinical Sciences, Section for Infection Medicine, Lund University, Skåne University Hospital, Lund, Sweden; 2 Division of Infectious Diseases and Center for Infectious Medicine, Karolinska Institutet at Karolinska University Hospital Huddinge, Stockholm, Sweden; The Ohio State University, UNITED STATES

## Abstract

**Background:**

Increased body temperature in the Emergency Department (BT-ED) and the ICU (BT-ICU) is associated with lower mortality in patients with sepsis. Here, we compared how well BT-ED and BT-ICU predict mortality; investigated mortality in various combinations of BT-ED and BT-ICU, and; compared degree of fever in the ED and ICU and associated quality of care.

**Methods:**

2385 adults who were admitted to an ICU within 24 hours of ED arrival with severe sepsis or septic shock were included.

**Results:**

Thirty-day mortality was 23.6%. Median BT-ED and BT-ICU was 38.1 and 37.6°C. Crude mortality decreased more than 5% points per°C increase for both BT-ED and BT-ICU. Adjusted OR for mortality was 0.82/°C increase for BT-ED (0.76–0.88, p < 0.001), and 0.89 for BT-ICU (0.83–0.95, p<0.001). Patients who were at/below median temperature in both the ED and in the ICU had the highest mortality, 32%, and those with over median in the ED and at/below in the ICU had the lowest, 16%, (p<0.001). Women had 0.2°C lower median BT-ED (p = 0.03) and 0.3°C lower BT-ICU (p<0.0001) than men. Older patients had lower BT in the ICU, but not in the ED. Fever was associated with a higher rate of sepsis bundle achievement in the ED, but lower nurse workload in the ICU.

**Conclusions:**

BT-ED was more useful to prognosticate mortality than BT-ICU. Despite better prognosis in patients with elevated BT, fever was associated with higher quality of care in the ED. Future studies should assess how BT-ED can be used to improve triage of infected patients, assigning higher priority to patients with low-grade/no fever and vice versa. Patients with at/below median BT in both ED and ICU have the highest mortality and should receive special attention. Different BT according to sex and age also needs further study.

## Background

Severe sepsis and septic shock carry high mortality, particularly among patients who require intensive care [1]. We have previously shown that elevated body temperature (BT) in the emergency department is associated with better survival and shorter length of stay in patients admitted to the ICU because of community acquired severe sepsis or septic shock [2]. Similarly, mortality decreases with increasing BT in diverse hospital populations with bacteremia [3–6]. In the ICU, elevated maximum BT during the first 24 hours after ICU-admittance is associated with lower mortality in infected patients [7]. BT is thus of prognostic interest in critically ill septic patients, but it is unclear which is the optimal time point to measure–early, on ED admittance (BT-ED), or later, on ICU-admittance (BT-ICU). Whether combining BT-ED and BT-ICU measurements can yield further prognostic information is also unknown.

Furthermore, we have shown that increased BT is associated with better and more timely care of septic patients in the ED, despite worse prognosis in those with low BT [2]. Whether preferential treatment is also given in the ICU to those with elevated BT has not been studied to our knowledge.

Given these knowledge gaps, we compared the value of BT-ED and BT-ICU for predicting 30-day mortality; investigated how mortality varied in different combinations of BT-ED and BT-ICU, and; compared how fever affects quality of care in the ED and in the ICU.

## Methods

### Study design

We conducted a cohort study using two prospectively compiled Swedish national quality registers; the National Quality Sepsis Registry (NQSR) [2] and the Swedish Intensive Care Registry (SIR) www.icuregswe.org [8], estimating 30-day mortality according to BT at admission in the ED and maximum recorded BT during one hour before, to one hour after ICU admittance. The NQSR comprises adult patients in Sweden, aged greater than 17 years, admitted for community acquired severe sepsis or septic shock to any of 42 ICUs within 24 hours of arrival to any of thirty-two EDs [9]. At present, SIR covers all general ICU's in Sweden but coverage has not been complete during the entire study period. NQSR registration started in 2007 and this report includes patients registered until December 2015. Data on NQSR patients also registered in SIR was then extracted from that register. Information on comorbidity was obtained from the Swedish National Inpatient Register [10] and the Swedish Prescribed Drug Register [11].

### Data collection and variables

#### National Quality Sepsis Registry (NQSR) and one-hour sepsis-bundle

Designated infectious disease (ID) specialists at each site screen hospital records for eligible patients whose data are entered into the NQSR database. In cases of multiple recorded vital parameters or lactate measurements in the ED, the first after hospital arrival was used. We screened variables for outliers and data errors. If available data precluded correction, entries were treated as missing values. If vital parameters were recorded from the ambulance, but missing from the Emergency Department (ED), the former was used and if iv fluids were administered in the ambulance but not in the ED, patients were considered treated with iv fluids within one hour. For didactic reasons, and to facilitate comparison with our previous study [2], body temperature (BT) was divided into four categories (<37, 37–38.29, 38.3–39.49, ≥39.5°C). We used the following threshold values: 37°C (98.6°F) for normal human BT [12]; 38.3°C (100.9°F), which is a common threshold for significant fever [13] and; 39.5°C (103.1°F) as an arbitrary cutoff for very high fever. The ED quality of care variables evaluated in this study are similar to those of the 2015 revised sepsis bundle promoted by the Surviving Sepsis Campaign (SSC) [14]; but according to Swedish recommendations, they are to be completed within one, rather than the three hours until recently permitted in the SSC guidelines. Although in Sweden there was originally no emphasis on achieving the recommendations in an all-or-none "bundled" fashion, we used the bundle concept to allow comparison with other studies. The one-hour sepsis-bundle advises the following to be performed within one hour: 1. Iv fluids, 2. Lactate (or base excess) measured, 3. Blood culture before antibiotics and 4. Antibiotics administered. If information on any component was missing, the sepsis bundle was treated as missing, except for lactate or base excess, for which missing values were interpreted as not measured. Treatment limitations were considered present if noted in either or both NQSR and SIR. Missing values for treatment limitations or incorrect antibiotics were considered negative.

#### SIR and SAPS3

NQSR was linked to other national registers, including SIR, using the unique personal identity number of all Swedish residents. National intensive care admissions are prospectively collected in SIR. Data, including those necessary for calculation of SAPS3 score, are entered and validated locally. They are then transferred to a central database and screened for errors and outliers and are, if necessary, returned for revalidation before acceptance. As originally described, missing values are assumed to be within normal limits [15]. The score is based on variables that describe patient characteristics before admission, circumstances of admission, and the presence and degree of physiologic derangement on ICU admission [15]. SAPS3-variables include: age; length of hospital stay before admission to the ICU; what department the patient arrives from; specific current or previous diagnoses; earlier treatments using vasoactive drugs; acute or planned admission; cause of ICU admittance; acute, planned or no surgery; acute infection on admission; measures of physiological disturbances recorded during one hour before to one hour after ICU admittance—bilirubin; maximum body temperature; creatinine, heart frequency, leukocytes, pH level, thrombocytes, systolic blood pressure and oxygenation (PaO2/FiO2 and PaO2) +/- mechanical ventilation; estimated Glasgow Coma Score (in most cases transformed from Reaction Level Scale 85 [16]). Since SAPS3 awards points for patients with BT-ICU < 35°C, we used t-SAPS3—a temperature-adjusted SAPS3—in which points for BT were subtracted in 90 patients with BT-ICU < 35°C—in analyses that included BT. For the same reason, we used a temperature and pneumonia-adjusted score—tp-SAPS3—in ancillary analyses in which pneumonia was analyzed along with other diagnoses.

#### Charlson comorbidity index

Charlson comorbidity index (CCI) was calculated using a combination of four sources: comorbidity recorded in NQSR and SIR; the Swedish Prescribed Drug Register and; data from the Swedish Patient Register. The Swedish Prescribed Drug Register covers data on dispensed drugs for the Swedish population since 2005. The Swedish Patient Register covers nationwide inpatient care since 1987, and non-primary outpatient care since 2001, and uses International Classification of Disease codes [10]. Since the SAPS3 score already contains several of the same comorbidities, and to avoid over-adjustment, we calculated a modified CCI for multi-variate analyses in which both scores were used—points for the following comorbidities were subtracted: AIDS, hematological cancer, non-metastasized tumor, chronic liver failure (but not mild liver disease) and congestive heart failure.

#### Nursing workload scores

Three scoring systems have been used in SIR to register nursing workload during the time of this study:

1. Nine Equivalents of Nursing Manpower Use (NEMS) Score. It assigns points for nine activities, all relating to specific organ support, nursing, diagnostic and therapeutic interventions inside or outside the ICU, specifically: basic monitoring; intravenous medication; mechanical ventilatory support; supplementary ventilatory care; single vasoactive medication; multiple vasoactive medication; dialysis techniques; specific interventions in the ICU, or outside the ICU [17].
2. Nursing Care Recording System (NCR-11)
  Assigns points for nursing workload within 11 areas: basic monitoring; neurology; respiration; circulation; care for wounds, drains, stomies; nephrology; infusions/transfusions/enteral feeding; sampling (blood and other body fluids); hygiene and mobilization; special treatments; relatives and external contacts [18].
3. NCR- 2014

Used since 2013. Similar to NCR-11, except circulation has been traded for patient-related administration.

Nursing workload scores were available for all but 2% of patients, but no individual score covered more than 56%. 46% of patients with NEMS scores also had NCR-11 scores, and 13% had NCR2014 scores. NCR-11 and NCR2014 scores did not overlap. We constructed a composite score built from quartiles of the nursing scores for the first 24 hours in the ICU. Since NEMS had better documentation in the literature, and covered most patients, we used it when available. When NEMS was missing, quartiles of NCR-11 or NCR-2014 were used.

#### Definition of severe sepsis and septic shock

In the NQSR, severe sepsis and septic shock were defined using a modification of the definition proposed by the American College of Chest Physicians/Society of Critical Care Medicine [19]. SIRS criteria were disregarded and severe sepsis and septic shock were diagnosed if patients had clinically suspected infection together with criteria for severe sepsis or septic shock listed below. Severe sepsis was diagnosed in patients with either hypotension (systolic blood pressure < 90 mmHg or mean arterial pressure < 70 mmHg); hypoperfusion (plasma lactate > 1 mmol/L above normal upper level or base excess ≤ -5 mEq/L), or; signs of acute reduction of organ perfusion (not related to primary septic focus or underlying chronic disease) as manifested by at least one of the following: a) acute deterioration of mental status; b) arterial hypoxemia (PaO2/FiO2 < 33 kPa (248 mm Hg), or < 27 kPa (203 mm Hg) (if the lung was the primary focus of infection) c) oliguria (urine production < 0.5 mL/kg/hr for 2 hrs); d) acute deterioration of liver function (S-bilirubin > 45 micromol/L, or S-alanine transaminase more than twice elevated above reference value); or e) recent coagulation abnormality (INR > 1.5, activated partial thromboplastin time > 60 seconds, or platelets < 100 x 10^9^/L). If previous function was unknown, a-e were assumed to have been normal prior to hospital admittance.

A diagnosis of septic shock required severe sepsis in addition to mean arterial pressure < 70 mm Hg for > 30 mins despite adequate fluid resuscitation or hypotension demanding vasopressor support. Definite focus of infection was determined after discharge on the basis of complete medical records including cultures, imaging studies and laboratory tests. Correctness of administered antibiotics was determined by susceptibility testing or, in culture-negative cases, in relation to guidelines for empiric therapy.

#### Treatment restrictions

Treatment restriction was defined as a decision restricting to any extent current or future care including cardiopulmonary resuscitation, mechanical ventilation, dialysis, etc.

#### Cohort

**Fig 1:**
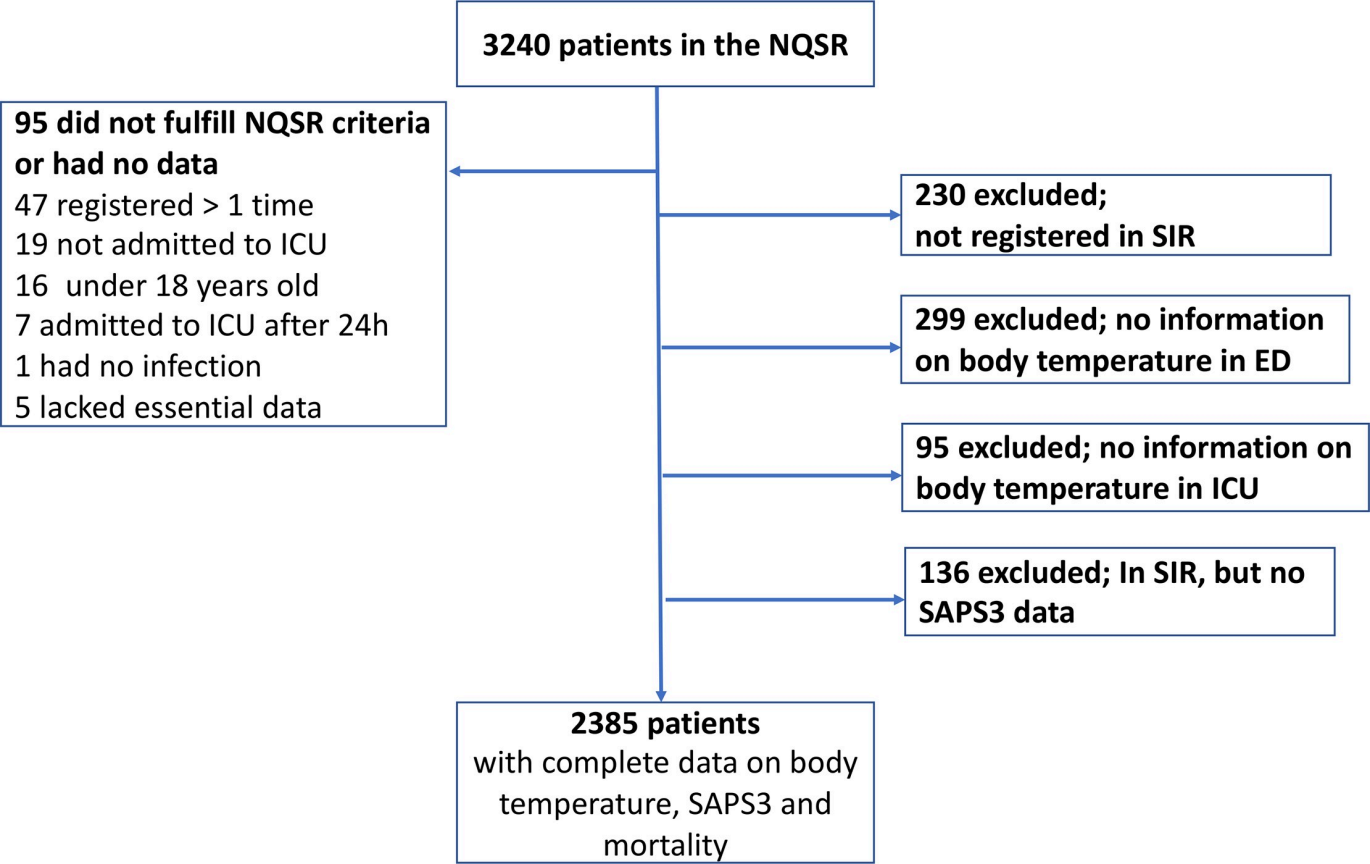
Ninety-five patients did not fulfill inclusion criteria or lacked essential data. 230 were were not registered in SIR. 299 lacked recorded body temperature in the ED and 95 lacked recorded maximum temperature around ICU admittance. A final 136 patients were excluded since a different severity score was used in the ICU and they lacked SAPS3 data.

Three thousand two hundred forty patients were recorded in the NQSR by December 2015. 855 were excluded, leaving 2385 for the main analyses, Fig 1.

Patients were admitted via 32 EDs to 42 ICUs. 21% had missing complete values for the composite factor 1-hour sepsis bundle. Missing physiological values for the SAPS3 score varied from 0% for some vital parameters up to 12% for individual lab tests–overall, SAPS3 was calculated based on complete physiological variables in 81% of cases. A composite nursing workload score could be calculated for all but 2% of subjects.

#### Statistical methods

Chi square and Wilcoxon signed rank, and Kruskal-Wallis tests were used to assess the distribution of risk factors for 30-day mortality between strata of BT in the ED and in the ICU. Using logistic regression, we estimated the odds ratios (ORs) for 30-day mortality according to BT-ED and BT-ICU. We used generalized estimating equations (GEEs) to account for the possibility of dependency between individuals admitted through the same ED. The covariates were first examined separately in univariate models, and then in multivariate models. The area under the receiver-operating characteristics (ROC) curves were compared to assess the capacity of BT-ED and BT-ICU to predict 30-day mortality. SAPS3 is constructed for global risk adjustment and was used in the main multivariate model alongside sex and quality of care measures. Since we wanted to adjust as thoroughly as possible for potential confounders to the temperature-mortality association, we added two important risk factors that are not accounted for in SAPS3 –treatment restrictions and incorrect antibiotics. We performed ancillary analyses assessing the main model A) subtracting incorrect antibiotics and treatment limitations, and B) adding comorbidities and discharge diagnoses not included in calculation of the SAPS3 score. Stata version 15 was used for statistical analyses (StataCorp, College Station, TX).

#### Ethics approval and consent to participate

Study approval was granted by the ethical review board in Stockholm (2015/901-32). Written informed consent was waived.

## Results

The final cohort included 2385 patients with complete data on BT-ED, BT-ICU, SAPS3 scores and 30-day mortality. Fifty-six percent were male and median age was 68 (interquartile range [IQR], 57–77). 30-day mortality was 23.6% and median length of stay (LOS) of survivors was 13 days (IQR, 8–25). Pneumonia was diagnosed in 830 patients (34.8%), urinary tract infection in 497 (20.8%), abdominal infection in 251 (10.5%), other foci in 508 (21.3%) and unknown focus in 265 (11.1%). Data on infectious focus was missing for 34 patients (1.4%). Patients were admitted to 42 ICU’s; 773 in seven tertiary (university) hospitals and 1,612 patients in 25 secondary (county) hospitals.

### BT-ED, BT-ICU and mortality

**Fig 2:**
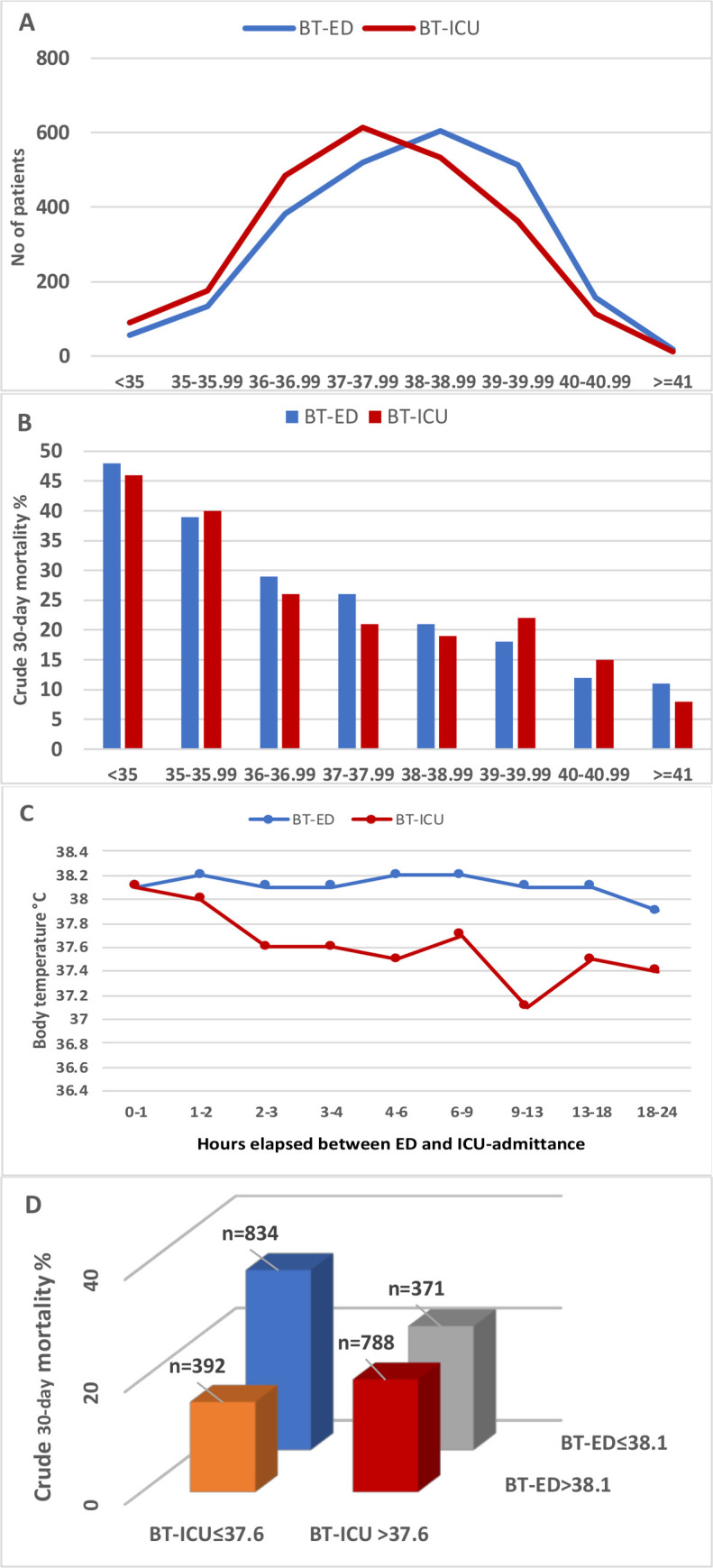
**A)** Distribution of patients per body temperature in the ED and in the ICU. **B)** crude 30-day mortality using BT-ED and BT-ICU. **C)** BT-ED and BT-ICU according to time elapsed between ED and ICU admittance. **D)** Crosswise comparison of crude 30-day mortality in patients over and under median body temperature in the emergency department and the ICU (p<0.001). BT-ED body temperature in the Emergency Department. BT-ICU body temperature in the ICU. 35°C = 95°F, 36°C = 96.8°F, 37°C = 98.6°F, 38°C = 100.4°F, 39°C = 102.2°F, 40°C = 104°F, 41°C = 105.8°F.

Median BT-ED and BT-ICU was 38.1 (IQR 37.0–39.1) and 37.6°C (IQR 36.6–38.7) (100.6 and 99.7°F), respectively, Fig 2A. Crude 30-day mortality fell on average 5% points per°C increase, for both BT-ED and BT-ICU, from 35 to > 41°C (95.0 to 105.8°F), Fig 2B. OR for mortality per°C increase was 0.78 (0.75–0.82; *p* < 0.001) for BT-ED, and 0.82 (0.78–0.86; *p* < 0.001) for BT-ICU in unadjusted analysis, and 0.82 (0.76–0.88, p < 0.001) for BT-ED and 0.89 (0.83–0.95, p<0.001) for BT-ICU in an analysis adjusted for t-SAPS3, sex, sepsis bundle completion, treatment limitations and incorrect antibiotics. The area under the ROC curve for mortality was 0.60 for BT-ED vs 0.59 for BT-ICU (p = 0.21). Median time elapsed between ED and ICU admittance was 3 hours and 23 minutes. Patients transferred within the first two hours had similar median BT-ED and BT-ICU, whereas those transferred later had on average 0.5°C lower BT-ICU throughout the remainder of the twenty-four hour inclusion window, Fig 1C.

Table 1 shows clinical outcomes and the distribution of risk factors for 30-day mortality by temperature strata for BT-ED and BT-ICU. Mortality decreased for both with increasing temperatures, as did length of stay (LOS) of survivors in both ICU and hospital. Age, sex and Charlson comorbidity index were equally distributed for BT-ED, but differed significantly for BT-ICU: patients in the lowest ICU temperature category were a median six years older than those in the highest; females constituted almost 51% in the lowest vs 35% in the highest, and; Charlson comorbidity index was a mean 0.3 points higher in the lowest compared to the highest category. SAPS3 scores were higher in lower BT-ED and BT-ICU strata, and treatment limitations were more common.

**Table 1:** Distribution of outcomes, demographic factors and risk factors for 30-day mortality by strata of temperature measured in the ED and in the ICU.

Characteristic	<37°C	37–38.29°C	38.3–39.49°C	≥39.5°C)	p-value ^*b*^
**BT-ED, n**	572	692	744	377	
**BT-ICU, n**	750	814	563	258	
***Patient outcomes***					
**30-d Mortality BT-ED**	33.0	24.1	20.0	15.4	**<0.001**
**30-d Mortality BT-ICU**	31.5	21.3	19.2	17.8	**<0.001**
**H-LOS, surv, BT-ED, d** ^**c**^	18, 9–34	14, 8–27	12, 7–22	12, 7–21	**0.0001**
**H-LOS, surv, BT-ICU** ^**c**^	16, 8–30	14, 8–24	12, 7–24	12, 6–22	**0.002**
**ICU-LOS s BT-ED, hours**^**c**^	71 (41–149)	54 (26–144)	50 (25–112)	46 (24–112)	**0.0001**
**ICU-LOS surv BT-ICU, h**^**c**^	65 (29–146)	54 (26–122)	49 (26–119)	45 (23–110)	**0.003**
***Demography***					
**Age BT-ED** ^**c**^	68, 56–76	68, 56–77	69, 58–78	68, 55–76	0.14
**Age BT-ED, male** ^**c**^	67, 56–75	68, 56–76	68, 59–78	70, 59–78	0.12
**Age BT-ED, female** ^**c**^	69, 57–78	68, 56–77	69, 57–78	65, 49–75	**0.02**
**Age BT-ICU** ^**c**^	70, 60–78	68, 56–77	67, 55–76	64, 51–74	**0.0001**
**Age BT-ICU, male** ^**c**^	69, 71–77	70, 58–78	67, 55–76	65, 54–74	**0.001**
**Age BT-ICU, female** ^**c**^	71, 59–79	66, 54–76	67, 55–76	63, 43–75	**0.0001**
**Sex BT-ED, % female** ^**c**^	45.8	46.1	42.1	41.6	0.27
**Sex BT-ICU- % female** ^**c**^	50.7	44.7	38.4	35.3	**<0.001**
***Comorbidity***					
**Charlson CI BT-ED**	2, 1–4 ^d^ (2.34±2.25 ^c^)	2, 1–4 ^d^ (2.33±2.22^c^)	2, 1–4 ^d^ (2.40±2.21 ^c^)	2, 1–3 ^d^ (2.23±2.33 ^c^)	**0.28**
**Charlson CI BT-ICU**	2, 1–4 ^d^ (2.48±2.3 ^c^)	2, 0–3 ^d^ (2.20±2.18 ^c^)	2, 1–4 ^d^ (2.43±2.23 ^c^)	2, 0–3 ^d^ (2.21±2.22 ^c^)	**0.04**
***Severity score***					
**SAPS3 BT-ED** ^**c**^	67, 59–77	64, 56–73	63, 55–72	62, 55–70	**0.0001**
**SAPS3 BT-ICU** ^**c**^	67, 58–76	63, 55–72	63, 55–71	62, 53–73	**0.0001**
**t-SAPS3 BT-ED** ^**c**^	67, 58–76	64, 56–73	63, 55–72	62, 55–70	**0.0001**
**t-SAPS3 BT-ICU** ^**c**^	66, 58–76	63, 55–72	63, 55–71	62, 53–73	**0.0001**
***Quality of care in ED***					
**Compl sepsis bundle (%) (n = 1846) BT-ED** ^*a*^	27.2	31.6	43.1	48.0	**<0.001**
**Incorrect AB BT-ED (%)**	5.8	8.5	7.9	7.7	0.30
***Resource utilization in ICU***					
**Composite NWS BT-ED(n = 2337)** ^**c**^^,^^**d**^	3 (1–4) 2.50 (1.17)	2 (1–3) 2.36 (1.13)	2 (1–3) 2.28 (1.28)	2 (1–3) 2.24 (1.14)	**0.004**
**Composite NWS BT-ICU (n = 2337)** ^**c**^^,^^**d**^	3 (1–3) 2.47 (1.18)	2 (1–3) 2.36 (1.14)	2 (1–3) 2.21 (1.11)	2 (1–3) 2.22 (1.14)	**0.004**
**Ventil BT-ED (n = 2374)**	32.1	34.0	27.4	24.3	**0.002**
**Ventil BT-ICU (n = 2374)**	33.9	27.9	28.6	27.0	**0.04**
***Treatment limitations***					
**at 48h BT-ED (%)**	23.6	20.1	19.5	14.6	**0.009**
**at 48 h BT-ICU (%)**	26.1	18.4	16.5	13.6	**<0.001**

37°C = 98.6°F, 38.29°C = 100.92°F, 39.49°C = 103.1°F, AB: Antibiotics, BT-ED: Body temperature measured in Emergency Department, BT-ICU: Body temperature measured in Intensive Care Unit, Compl sepsis bundle: Complete sepsis bundle, all four components initiated within 1h, H-LOS, surv, d: Hospital length of stay survivors, days, ICU-LOS s, h: ICU length of stay survivors, hours, Charlson CI: Charlson Comorbidity Index, NWS: Nursing Workload Score, SAPS3: Severe Acute Physiology Score 3, t-SAPS3: SAPS3 minus temperature score for patients with body temperature < 35°C (n = 90). Ventil: Ventilator

^*a*^ years 2009–2015 (excluding 2007–2008)

^*b*^ for difference between temperature categories

^**c**^ Median (IQR)

^d^ Mean (SD)

**Fig 3:**
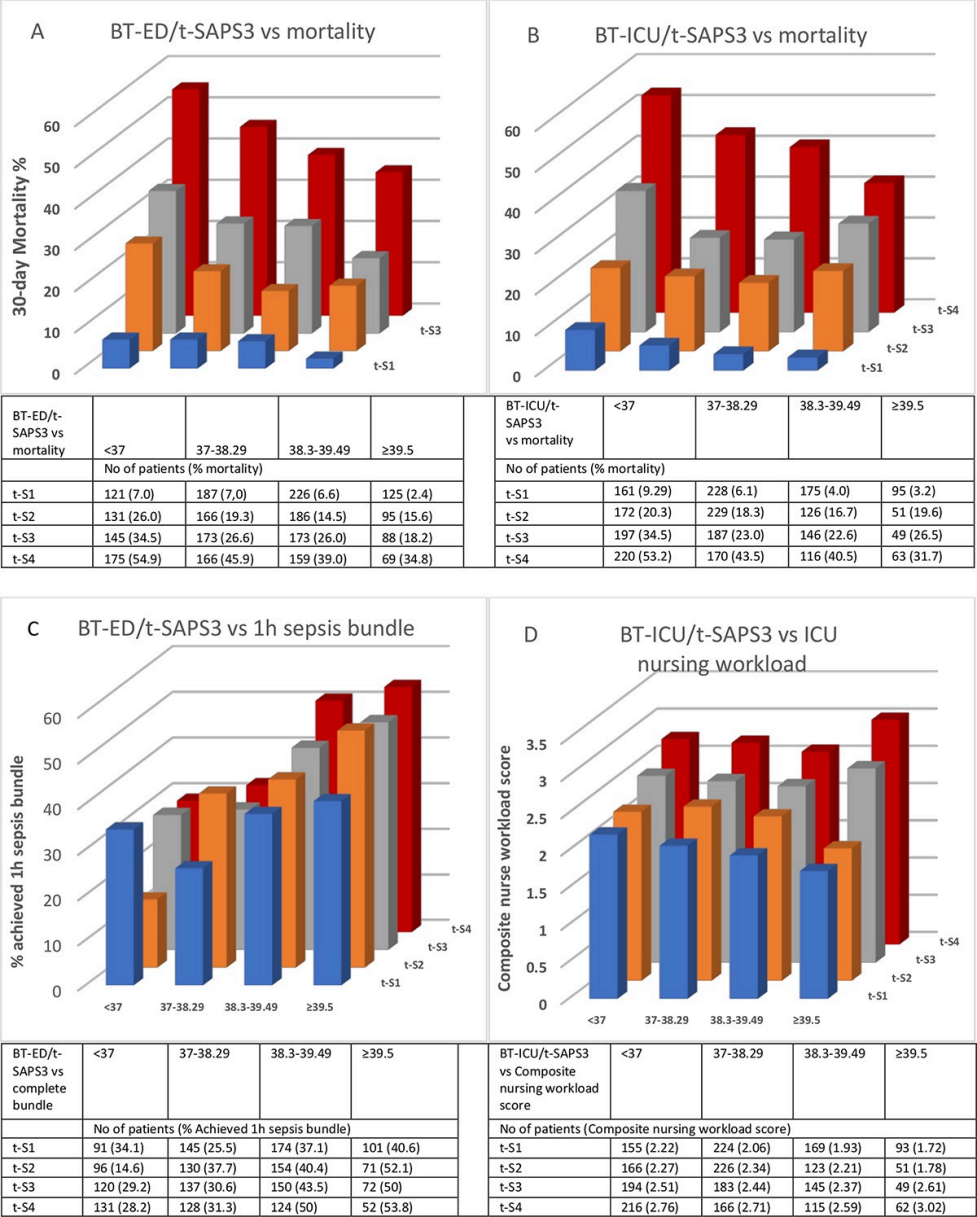
Body temperature in the ED (**A** and **C**) and the ICU (**B** and **D**) stratified by quartiles of t-SAPS3 (t-S1 lowest quartile) versus 30-day-mortality (**A** and **B**), achieved one-hour sepsis bundle (all four components) (**C**) and composite ICU nursing workload score (**D**). BT-ED: body temperature in the Emergency Department, BT-ICU: body temperature in the ICU, SAPS3: Simplified Acute Physiology Score 3, t-SAPS: SAPS minus temperature score for patients with body temperature < 35°C (n = 90).

Despite higher severity of disease and mortality among patients with lower temperatures (Table 1, Fig 3A and 3B), the ratio of completed one-hour sepsis bundles was significantly higher in the ED among those with increased BT, Table 1, Fig 3C. In the ICU, by contrast, nurse workload and ventilator use were significantly higher in lower BT-ICU strata, Table 1, Fig 3D.

Table 2 shows the univariate ORs for individual risk factors and the ORs from multivariate analyses for 30-day mortality comparing BT-ED and BT-ICU. The strength of the association with mortality was stronger for BT-ED compared to BT-ICU in both adjusted and unadjusted analyses. Ancillary analyses presented in Table 3 show the main model with and without adjustment for incorrect antibiotics and with adjustment for comorbidities and diagnoses not accounted for in the SAPS3 score, all of which altered ORs for death only marginally.

**Table 2:** Odds ratios for death according to ED and ICU admission temperature ^a^.

	Uni-variate analysis (n = 2385)			Multi-variate analysis ^*b*^ BT-ED (n = 1846)			Multi-variate analysis ^*b*^ BT-ICU (n = 1846)		
Characteristic	OR	95% CI	p-value	OR	95% CI	p-value	OR	95% CI	p-value
***Demography***									
**Sex (female)**	1.15	0.95–1.40	0.16	1.28	0.98–1.67	0.07	1.26	0.98–1.64	0.08
***Severity of disease***									
***t-SAPS3 (per unit)***	**1.07**	**1.06–1.09**	**<0.001**	**1.06**	**1.05–1.08**	**<0.001**	**1.06**	**1.05–1.07**	**<0.001**
***BT as continuous variable*, *OR/°C increase***									
**BT-ED**	**0.78**	**0.75–0.82**	**<0.001**	**0.82**	**0.76–0.88**	**<0.001**			
**BT-ICU**	**0.82**	**0.78–0.86**	**<0.001**				**0.89**	**0.83–0.95**	**<0.001**
***Quality of care***									
**Bundle1h** ^*c*^ **(n = 1846)**	1.06	0.85–1.33	0.60	1.19	0.93–1.52	0.17	1.09	0.88–1.38	0.45
**Incorrect AB**	1.47	0.97–2.23	0.07	**1.99**	**1.15–3.44**	**0.01**	**1.89**	**1.11–3.20**	**0.02**
***Treatment limitations***									
**at 48 h**	**10.95**	**8.79–13.64**	**<0.001**	**7.80**	**6.01–10.11**	**<0.001**	**7.52**	**5.80–9.76**	**<0.001**

AB: Antibiotic. BT: Body temperature. ED: Emergency Department. ICU: Intensive Care Unit. SAPS3: Simplified Acute Physiology Score 3. t-SAPS3: SAPS3 minus points for temperature. Treatment limitations: Any treatment limitations ordered within 48 hours of admittance

^*a*^ estimated by GEE logistic regression

^*b*^ adjusted for all variables in the column

^*c*^ All Sepsis bundle components achieved within one hour. Significant risk factors in **bold text.**

**Table 3:** Odds ratios for death according to admission temperature, additional models ^a^.

	1 (n = 2385)		2 ^*b*^ (n = 1846)	3 ^*b*^ (n = 1846)	4 ^*b*^ (n = 1846)	5 ^*b*^ (n = 1846)	6 ^*b*^ (n = 1846)	7 ^*b*^ (n = 1846)
Character-istic	OR (95% CI), p-value		OR (95% CI), p-value	OR (95% CI), p-value	OR (95% CI), p-value	OR (95% CI), p-value	OR (95% CI), p-value	OR (95% CI), p-value
***Demography***								
**Sex (female)**	1.15 (0.95–1.40), 0.16		1.28 (0.98–1.67), 0.07	1.26 (0.98–1.64), 0.08	1.25 (0.97–1.62), 0.09	1.24 (0.96–1.59), 0.10	1.27 (0.96–1.67), 0.09	1.27 (0.97–1.66), 0.09
***BT as continuous variable*, *OR/°C increase***								
**BT-ED**	**0.78 (0.75–0.82), <0.001**		**0.82 (0.76–0.88), <0.001**		**0.83 (0.77–0.89), <0.001**		**0.83 (0.77–0.90), <0.001**	
**BT-ICU**	**0.82 (0.78–0.86), <0.001**			**0.89 (0.83–0.95), <0.001**		**0.88 (0.82–0.94), <0.001**		**0.90 (0.84–0.96), 0.001**
***Severity of disease***								
**SAPS3 (per unit)**	**1.07 (1.06–1.09), 0.001**							
**t-SAPS3**	**1.07 (1.06–1.09), <0.001**		**1.06 (1.05–1.08), <0.001**	**1.06 (1.05–1.07), <0.001**	**1.07 (1.06–1.09), <0.001**	**1.07 (1.06–1.09), <0.001**		
**tp-SAPS3**	**1.07 (1.06–1.08), <0.001**						**1.06 (1.05–1.08), <0.001**	**1.06 (1.05–1.08), <0.001**
***Underlying co-morbidity***								
**Charlson modified**^**c**^	**1.09 (1.03–1.16), 0.003**						0.98 (0.90–1.07), 0.63	0.98 (0.90–1.07), 0.65
***Diagnosis***								
	**OR (CI)**						**OR (CI)**	**OR (CI)**
**Pneumonia**	**1.00 (*Ref*.*)***						**1.00 (*Ref*.*)***	**1.00 (*Ref*.*)***
**Urinary tract**	**0.60 (0.44–0.83)**						**0.43 (0.26–0.71)**	**0.40 (0.25–0.66)**
**Abdominal**	**1.59 (1.20–2.12)**						**0.95 (0.61–1.47)**	**0.96 (0.62–1.48)**
**Other focus**	**0.81 (0.59–1.11)**						**0.75 (0.47–1.21)**	**0.73 (0.45–1.18)**
**Unknown focus**	**1.90 (1.48–2.45)**						**1.18 (0.81–1.72)**	**1.14 (0.78–1.65)**
**p-value**	**<0.0001**						**0.006**	**0.003**
***Quality of care***								
**Sepsis Bundle 1h (n = 1846)**		1.06 (0.85–1.33), 0.60	1.19 (0.93–1.52), 0.17	1.09 (0.88–1.38), 0.45	1.13 (0.92–1.40), 0.25	1.06 (0.86–1.29), 0.59	1.19 (0.95–1.50), 0.14	1.11 (0.89–1.40), 0.35
**Incorrect antibiotic**	1.47 (0.97–2.23), 0.07		**1.99 (1.15–3.44), 0.01**	**1.89 (1.11–3.20), 0.02**			**2.05 (1.20–3.49), 0.008**	**1.95 (1.16–3.28), 0.01**
**Treatment limitations**	**10.95 (8.79–13.64), <0.001**		**7.80 (6.01–10.11), <0.001**	**7.52 (5.80–9.76), <0.001**			**8.10 (6.41–10.22),<0.001**	**7.86 (6.27–9.98), <0.001**

BT: Body temperature, ED: Emergency department, SAPS3: Simplified Acute Physiology Score. t-SAPS: SAPS minus temperature score for patients with body temperature < 35°C (n = 90), tp-SAPS3: SAPS3 minus points for temperature and pneumonia, CLF: Chronic Liver Failure, CHF: Congestive Heart Failure, COPD: Chronic Obstructive Pulmonary Disease, CKF: Chronic Kidney Failure.

^*a*^ Estimated by generalized estimating equations, logistic regression.

^*b*^ Models: 1 Univariate; 2–7 multivariate, adjusted for all other variables in the column; 2 and 3 Main model, same as in Table 2; 4 and 5 Main model (Table 2) minus Incorrect antibiotics and Treatment limitations; 6 and 7 Main model (Table 2) plus chronic diseases not included in SAPS3 and final infectious diagnosis, tp-SAPS3 used instead of t-SAPS3.

^**c**^ Minus comorbidities already included in SAPS3 score. Significant risk factors in **bold text.**

### Combining BT-ED and BT-ICU

Crosswise combinations of the ED and ICU groups showed a higher mortality, 32%, in those who had BT at median or below in both the ED and in the ICU, Fig 2C. The lowest mortality, 16%, was found in the group with a combination of above median BT in the ED and at/below in the ICU, whereas those with above median temperature at both measurements had a mortality of 20% (p<0.001 for all groups). Fig 3A and 3B show how the relationship between mortality and BT-ED/BT-ICU remained similar when stratified by severity of disease.

### Gender and age

Since age and gender distribution were skewed across BT-ICU strata, we analyzed these characteristics further. We found that women had 0.2°C lower median temperature in the ED (p = 0.03) and 0.3°C in the ICU (p<0.0001). Across BT-ED strata, there was no significant difference in the proportion of women in the highest and lowest temperature categories, but across BT-ICU strata, the proportion of women fell significantly with increasing temperatures, Table 1.

In a combined analysis of age and gender, we found that women in the lowest BT-ICU strata were a median eight years older than those in the highest compared to four years for men. Across BT-ED strata, there was no significant age difference for men, but women in the lowest temperature category were four years older than those in the highest. In our main model (adjusting for severity of disease, quality of care, incorrect antibiotics and treatment limitations), OR for mortality per°C increase was 0.83 (0.72–0.94; *p* = 0.005) for BT-ED among men and 0.81 (0.72–0.90; *p* < 0.001) for women. For BT-ICU, the corresponding OR’s were 0.90 (0.79–1.03; *p* = 0.13) for men and 0.88 (0.78–0.99; *p* = 0.04) per degree Celsius increase in women.

## Discussion

### Main findings

This large, national, multi-center study using two independent national registers is the first to compare the prognostic significance of ED vs ICU admission body temperature in community acquired sepsis. We found that: 1) elevated body temperature on ED and ICU admission of patients with severe sepsis or septic shock predicted increased survival, but that BT-ED has a stronger association with mortality and is therefore of greater prognostic use; 2) temperature at/below median in both ED and the ICU was associated with the highest mortality; 3) despite better prognosis, patients with fever obtained higher sepsis bundle achievement in the ED, but incurred lower nurse workload in the ICU; 4) older patients had lower BT in the ICU, but not in the ED; 5) women had lower BT than men.

### BT-ED vs BT-ICU for predicting death

We confirmed our previous finding of a strong and linear association between increased BT-ED and survival [2] which remained consistent after adjustment for severity of disease as measured by SAPS3, a widely used severity score [15]. Our results also support a previous report [7] that increased maximum BT in the ICU is associated with 30-day survival, and shorter ICU and hospital LOS.

In our study, the strength of the association between mortality and BT was stronger for BT-ED than for BT-ICU, although there was no significant difference between the areas under the ROC curve in the crude analysis. Median BT-ICU was 0.5°C lower than BT-ED, which probably explains the difference. The temperature difference was only seen in those who waited at least two hours after ED- until ICU-admission, after which point median BT-ICU remained steadily around 0.5°C lower throughout the inclusion period. Fever thus decreases over time. Whether this is explained by spontaneous resolution of fever, by sepsis bundle achievement, which is higher in those with increased body temperature, and includes time to antibiotics and iv fluids, or can be attributed to other sepsis-related therapy including antipyretic drugs before arrival to the ICU (not recorded in the registers), or by external factors such as cold rooms, is unclear.

As expected, patients who were at/below median temperatures in both the ED and the ICU had the highest mortality (32%). Since higher BT-ED and BT-ICU were independently associated with better survival, it was surprising to find that the lowest mortality was found not in patients who were above median at both measurements (20%), but in those with a combination of above median temperature in the ED and at/below in the ICU (16%). The difference, however, was not large and the latter group was relatively small (n = 392). It may also contain a sizeable fraction of patients with robust initial fever *and* an early response to treatment.

Possible pathophysiological explanations for the positive association between fever and survival include fever causing: negative feedback on secretion of pyrogenic cytokines; improved function of immune cells; better effect of antibiotics due to reduced minimal inhibitory concentration (MIC) against bacteria and; inhibition of bacterial and viral replication [20, 21]. Lack of temperature increase or hypothermia, on the other hand, may be explained by a weak immune response; alternatively hypothermia may be an adaptive response in severe illness, during which sepsis-induced hypoxia may cause cells and organs to go into hibernation in order to conserve energy [22].

### BT and SAPS3

SAPS3 was developed expressly to predict mortality, is calculated based on a large number of variables, and normally makes accurate stand-alone predictions. This study shows that BT adds considerable and independent predictive value in critically ill septic patients.

### BT and quality of care

We have previously shown that increased BT was associated with more timely and better quality of care in the ED [2], subsequently confirmed in other studies [23, 24], despite lower mortality in patients with increased BT. Here, we show that the association remains consistent, after stratification for severity using SAPS3. In the ICU, by contrast, nursing workload and ventilator use was higher for lower BT-ICU strata. Presumably, once patients are hooked up in the ICU, other prognostic information outweighs the presence or absence of fever.

### BT and age

An interesting finding was that age distribution differed between categories of temperature measured in the ED and in the ICU; in the ED, median age was the same across temperature categories whereas in the ICU, median age was six years higher in the lowest compared to the highest temperature category, raising the question whether elderly patients have trouble sustaining fever over time, or respond more readily to therapy. The finding agrees with several previous studies which have found lower BT among infected elderly patients [25–27]. We found no literature that has explored persistence of fever during sepsis in the elderly.

### BT and sex

Some surprising results related to sex; first, women had lower median temperature in the ED and in the ICU. Second, across BT-ED strata, there was no significant difference in the proportion of women in the highest and lowest temperature categories, but across BT-ICU strata, the proportion of women fell significantly with increasing temperatures. We found no studies that explicitly set out to study gender differences in fever, but did find two in which women with infections had lower temperatures than men [28, 29] and none that showed higher, or equal temperatures.

Combining age and gender, we found that an age gradient across temperature categories was steeper among women than men for both BT-ED and BT-ICU so that women in the lowest temperature categories were four and eight years older respectively than in the highest categories. This may indicate that old women have more trouble producing and maintaining fever than old men. Despite differences in temperature response, OR for mortality per degree Celsius increase was similar in both sexes.

### Strengths and weaknesses

Our study has several strengths. It is population-based and is well-powered to compare variations in mortality and treatment imbalances according to temperature subgroups. It drew information from national registers which has enabled an unbiased nationwide identification of patients with severe sepsis and septic shock. A particular strength is the independent association with survival of both BT-ED and BT-ICU, which were measured as part of clinical routine in different departments, at different time points and in different circumstances. A major shortcoming of our previous study on ED temperature and mortality [2] was the lack of an established severity of disease score in the adjusted analyses. Here, we could amend that weakness using SAPS3-score to adjust for severity. Physiological data underlying SAPS3 calculations were complete in 81% of cases. This is better than a general Swedish ICU-cohort, in which only 59% of patients had complete variables, but for which SAPS3 was deemed to perform well [30]. Even though SAPS3 is calculated around ICU admittance, up to twenty-four hours after arrival to the hospital, we believe that it is the best available source in our data for estimating illness severity in the ED. The time frame allowed between ED and ICU admittance means that time elapsed between ED and ICU BT measurements was anywhere between 0 and 25 hours, since maximum BT-ICU was measured from one hour before to one hour after ICU-admittance.

A remaining weakness is the lack of data on fever-modifying therapy, wherefore residual confounding cannot be ruled out. And although analyses were adjusted for quality of care, it is possible that the temperature-mortality association was related to other differences in treatment not captured in the databases. Also, the NQSR does not capture all eligible patients in Sweden, since it only comprises hospitals where ID physicians are present, and registration rates vary between centers [9].

## Conclusions

Fever on ED and ICU admission was strongly associated with decreased mortality in critically ill septic patients in both crude and adjusted analyses. BT measured in the ED was better than maximum BT around ICU admittance for prognosticating 30-day mortality. Despite less severe disease and ultimately lower mortality in patients with fever, those patients received better care in the ED, but not in the ICU, indicating more adequate patient assessment in the ICU. Future studies should investigate how BT-ED can be used to improve management of infected patients in the ED, specifically by modifying current triage models to assign higher priority to those with low BT, and vice versa. Patients with persistently below median temperature in both the ED and in the ICU have the highest mortality and should receive special attention. Women had lower BT than men, and older patients, particularly women, had trouble sustaining fever—findings that merit further study.
